# Interface Characterization Between Polyethylene/Silica in Engineered Cementitious Composites by Molecular Dynamics Simulation

**DOI:** 10.3390/molecules24081497

**Published:** 2019-04-16

**Authors:** Shuai Zhou, Nam Vu-Bac, Behrouz Arash, Hehua Zhu, Xiaoying Zhuang

**Affiliations:** 1 College of Materials Science and Engineering, Chongqing University, Chongqing 400045, China; 2 Institute of Structural Mechanics, Bauhaus Universitat-Weimar, Marienstr 15, D-99423 Weimar, Germany; bac.nam.vu@uni-weimar.de; 3 Institute of Statics and Dynamics, Leibniz University Hannover, Hannover 30167, Germany; b.arash@isd.uni-hannover.de; 4 National Laboratory for Disaster Reduction in Civil Engineering, College of Civil Engineering, Tongji University, Shanghai 200092, China; zhuhehua@tongji.edu.cn

**Keywords:** self-healing concrete, autogenous healing, ECC, interface

## Abstract

Polyethylene is widely adopted in engineered cementitious composites to control the crack width. A clearer knowledge of the PE/concrete interfacial properties is important in developing engineered cementitious composites, which can lead to a limited crack width. Tensile failure and adhesion properties of the amorphous polyethylene/silica (PE/S) interface are investigated by molecular dynamics to interpret the PE/concrete interface. The influence of the PE chain length, the PE chain number and coupling agents applied on silica surface on the interfacial adhesion is studied. An increase of the adhesion strength of the modified silica surface by coupling agents compared with the unmodified silica is found. The failure process, density profile and potential energy evolutions of the PE/S interface are studied. The thermodynamic work of adhesion that quantifies the interfacial adhesion of the PE/S interface is evaluated. The present study helps to understand the interfacial adhesion behavior between ECC and PE, and is expected to contribute to restricting the crack width.

## 1. Introduction

Engineered cementitious composite (ECC) is a kind of high-performance fiber reinforced composite. It has a tight crack width due to the fiber bridging effect, which shows relatively high strain capacity about 5% while conventional concrete only has 0.01%. Polyethylene (PE) is firstly adopted to control the crack width in ECC [1,2,3]. Hence, a clearer knowledge of the PE/concrete interfacial properties is important since a better bonding between ECC and PE results in a smaller crack width. The autogenous healing effect is found in ECC, and is more obvious if the crack width is limited [4,5,6,7]. Concrete is a heterogeneous material, and silica (S) makes up more than 40 percent by weight of the concrete. Considering that silica has a defined chemical structure, it is often adopted to represent concrete [8]. Here, the PE/S interface is studied to interpret the PE/concrete interface. 

**Figure 1:**
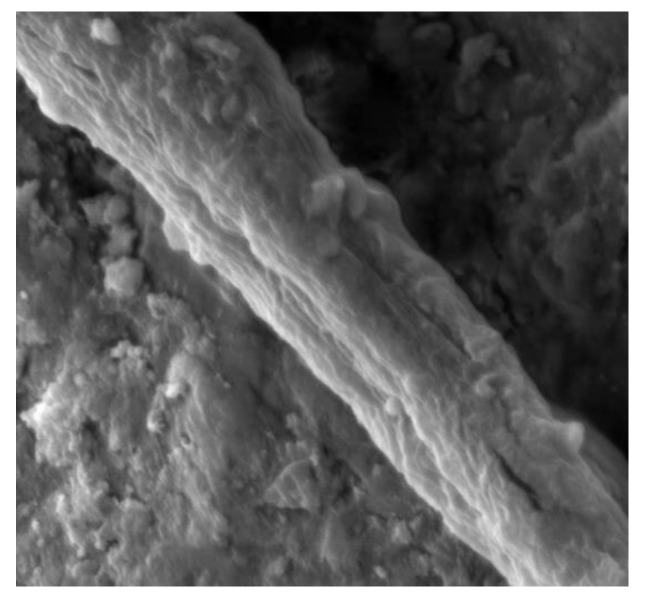
The necking of fibers in the cementitious matrix.

Molecular dynamics (MD) is an effective tool to study the interactions between two materials at the molecular level. There is various literature about interfaces [9,10,11,12,13,14,15,16,17]. The properties and the deformation mechanisms of the interfaces are explored. The PE/S interaction contains the shear component and the tensile component at microscales if the fiber is not perpendicular to the crack or not straight exactly. Meanwhile, the necking of fibers occurs during the pullout process, which also causes the tension between the fiber and the cementitious matrix due to the reduced cross-section of fibers by SEM tests (see Figure 1). The shear failure process of the PE/S interface is analyzed recently [18]. However, the tensile failure process and the interfacial bonding properties are neglected. The coupling agents [19] and surfactant [20] can enhance the bonding between the organic and inorganic materials. On one hand, the coupling agent has the reactive functional group that can form strong covalent crosslinks with polymers. On the other hand, it has the hydrolysable group capable of forming strong covalent bonds with the hydroxyl groups on the material surfaces [19]. There are many silane coupling agents like A151, A171, A172, etc. Only two typical silane coupling agents (i.e., A174 and A2783) are studied in this paper. MD simulations can reduce the number of trial-and-error steps in identifying the coupling agent and assist the selection process. The influence of coupling agents on the PE/S interface system under tension has not been investigated. 

In this study, the role of coupling agents in improving the interfacial adhesion and the fracture behavior of PE/S interfaces is investigated. Both neat SiO_2_ (S) and SiO_2_ modified with silane coupling agents (mS) are tested. Uniaxial tensile simulations are performed on the interface models. The influence of the chain length, the number of PE chains and the coupling agents on the interfacial adhesion is explored under tension. Internal mechanisms associated with chain movement are also investigated during the tension process. 

## 2. Details of the Simulation

### 2.1. MD Models

PE models (i.e., (C_2_H_4_)_n_) with 76 and 150 carbons of a chain are considered to study the influence of the chain length on the adhesion properties [21], and 20 and 40 chains are adopted to investigate the effect of the chain number on the adhesion properties [18]. The atomistic model is built by the Material Studio and relaxed using LAMMPS [22]. The Dreiding potential is utilized in PE, silica and the coupling agents [23] since it has been parameterized and validated for both organic and inorganic materials [24,25,26]. Related parameters of it come from previous research [23]. The cutoff distance is 12 Å, and the Verlet velocity algorithm is applied for integration with 1 fs time step, as is set in previous research [18]. The conjugate gradient algorithm is used to conduct the energy minimization [24]. After the model has been built, the system is equilibrated in the constant volume and constant temperature ensemble (NVT) using Nose-Hoover thermostat [27] at 600 K, and relaxed in the isothermal-isobaric ensemble (NPT) using Nose−Hoover barostat [28] at 600 K and atmospheric pressure, and then cooled down to 300 K [24]. The detailed model-building process has been illustrated in our previous research [18]. The densities of the equilibrated periodic PE boxes are in the range of 0.78–0.85 g/cm^3^, which agree with the experimental data [21]. The evaluated glass transition temperature (Tg) values for the PE models are between 232 K and 264 K, which agree with the experimental result of 190 K–300 K [29,30]. Hence, the stable PE models are achieved considering the corrected densities and Tg values [18].

**Figure 2:**
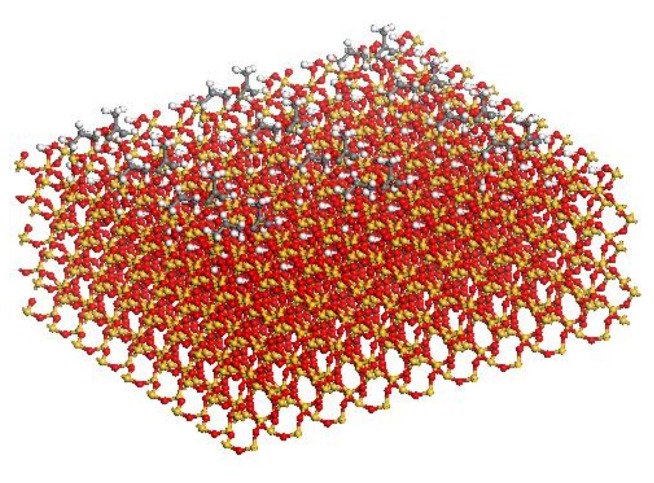
The model of mS.

Two typical types of silica coupling agents, A174 [31,32] and A2783 [33], are considered to study the influence of coupling agents here. A174 refers to γ-methacryloxypropyl trimethoxysilane (i.e., C_10_H_20_O_5_Si), and A2783 belongs to azidofunctional trialkoxysilane (i.e., C_14_H_31_O_3_N_3_Si). The chemical structures of A174 and A2783 are displayed in previous research [18], and the related coupling mechanism has be illustrated previously [33,34]. The silica (100) surface is adopted as the neat S [10]. A horizontal spacing around 0.5 nm between different coupling agents is used in the mS [19]. A174 has the hydrolysable group capable of forming strong covalent bonds with the hydroxyl groups on the material surfaces, while A2783 has the reactive functional group that can form strong covalent crosslinks with polymers and the hydrolysable group capable of forming strong covalent bonds with the hydroxyl groups on the material surfaces [19,33]. In the PE/mS models, the A174 attaches onto the silica via chemical covalent bonding [19], while the A2783 is connected with silica and PE chains by chemical covalent bonding [33]. Figure 2 shows silica (100) with the coupling agents. 

**Figure 3:**
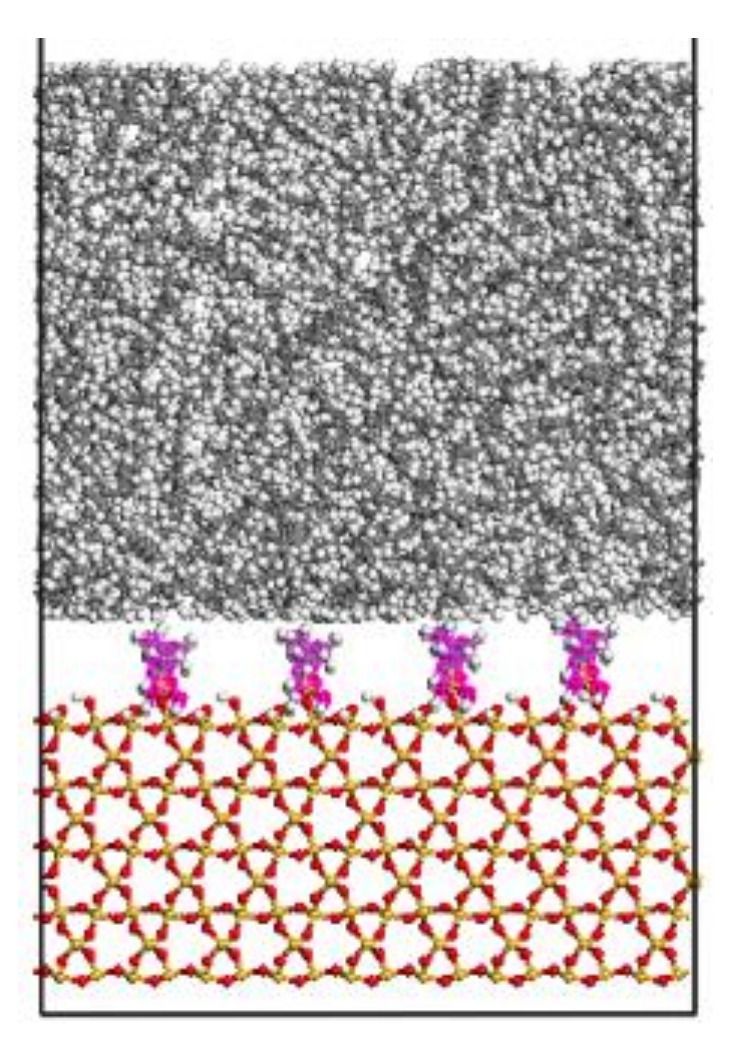
The molecular model of PE(76-40)/mS1.

The PE box is further extended to 1100 Å in the z-direction to reduce the interaction between different boxes in the 3D periodic boundary condition [9]. PE is built above S with a space of 0.5 nm [8]. The interface model is heated up to 500 K for 500 ps, and cooled to 300 K with a cooling rate of 0.2 K/ps, and then relaxed at 300 K for further 15 ns using the NVT ensemble. The average result for the last 1 ns is calculated for the interface energy. The PE(x − y)/S, PE(x − y)/mS1 and PE(x − y)/mS2 represent the equilibrated interface models of neat silica, A174-modified silica and A2783-modified silica combined with PE chains, respectively. Here, x is the chain length and y means the number of PE chains [18]. The PE(76-40)/mS1 interface model is exhibited in Figure 3.

### 2.2. Simulation of the Tensile Deformation of the Interfaces

**Figure 4:**
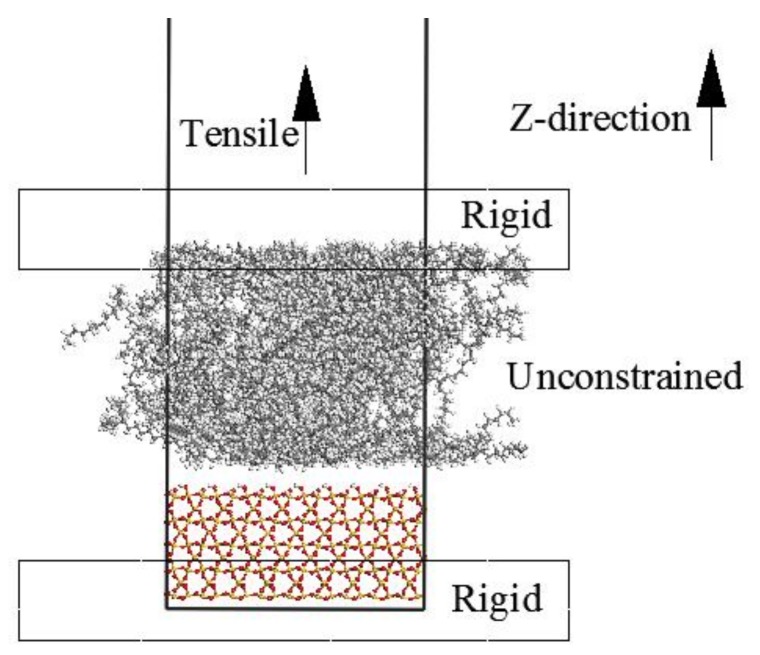
The PE(76-40)/S interface model during the tensile process.

The atoms of the top layer of PE as well as the bottom layer of S are kept fixed, while the middle part of PE and S is unconstrained, as displayed in Figure 4. The top frozen layer of the PE chains moves upwards along the z-direction with 1.0 Å at each step to simulate the interfacial separation. The middle unconstrained part is dynamically equilibrated for 1 ns using the NVT simulation at each step to obtain the normal stress using the virial theorem [9]. 

## 3. Results and Discussion

### 3.1. Thermodynamic Work of Adhesion

In order to investigate the effect of the modified silica on the adhesion behavior, it is required to evaluate the interfacial interactions by calculating the thermodynamic work of adhesion. The thermodynamic work of adhesion is directly related to the value of interaction energy according to the following equation [9]:

(1) $$W=\frac{-E_{inter}}{A}=\frac{E_{(m)S}+E_{PE}-E_{(m)S-PE}}{A}$$

where *A* is the contact area between the PE and S or mS surface. *E*_inter_, *E_(m)S_*, *E_PE_* and *E_(m)S-PE_* mean the interaction energy, the potential energy of S or mS, the potential energy of PE, the potential energy of the PE/S or PE/mS system, respectively.

The thermodynamic work of adhesion is obtained by using the molecular simulation of the energy change when two isolated surfaces come into close contact to create an interface [9,35]. The calculated thermodynamic work of adhesion averaged by 5 times with different initial configurations is listed in Table 1. With the larger chain length and chain number, the thermodynamic work of adhesion increases due to stronger interaction between PE and S or mS. Meanwhile, the thermodynamic work of adhesion between the PE and the mS grows greatly compared to the amount between the PE and S. In fact, the attachment of A174 to the S surface results in stronger dispersive forces and, consequently, greater interfacial interactions. At the same time, by using A174, the actual contact area between the PE and S increases. 

### 3.2. Tensile Elongation of the Interface

#### 3.2.1. Stress-Displacement Behavior

**Figure 5:**
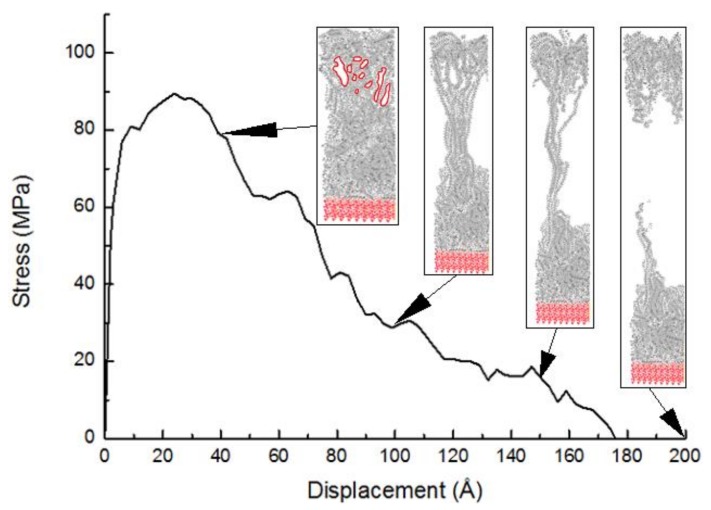
The tensile stress-displacement curve of the PE(150-40)/S interface and snapshots of tensile simulation at different displacements.

The tensile stress-displacement curve of PE(150-40)/S deformed at 300 K is exhibited in Figure 5. The curve at the low displacement or elastic region is ascending. After a maximum stress at the yield point, the stress is decreased due to raising the free volume needed for the atomistic motions under chain slipping, which shows a post-yield region. Finally, the stress becomes 0 and the failure happens. The MD simulations capture the detailed fracture process at the atomic scale. During the increase of the normal traction, the PE chains are stretched upward. As the normal strains increase, small voids quickly appear in the PE. The stress drop in Figure 5 around the separation displacements of 27 Å is caused by the separation among the PE chains. The separation is caused by the weak interaction of the van der Waals interactions acting between the chains. The post-yield behavior is exhibited by debonding between the chains, chain slipping, and disentanglement extending to an ultimate failure mode. The void formation is initiated in the regions of the polymer chains with weaker intermolecular interactions and less entanglement. The layering behavior may considerably change the physical properties of the polymer chains at the interface in comparison to the chains in the bulk region. The initial growing void is created far away from the interface layered zone, which is illustrated as a red circle in Figure 5. These voids become bigger and coalesce when the elongation further continues. The load transfer in graphene/carbon nanotube/PE hybrid nanocomposites via MD simulation was investigated and it was found that the first damage in PE/graphene interface was also observed in the PE bulk [36]. As the separation increases further, the void increases and the column becomes thinner at the displacement of 100 Å. At a separation of around 150 Å, a long and thin PE column emerges, connecting the top and bottom groups of the PE. Thereafter, the polymer chains are being pulled out from their starting configurations at the same time. The failure caused by the breakdown of nonbonded interactions in the PE bulk results in the cohesive failure mode at the displacement of 175 Å. As the disconnected chains in the column are attracted to the top and bottom groups, the column disappears, and the PE binder is separated by half. 

**Figure 6:**
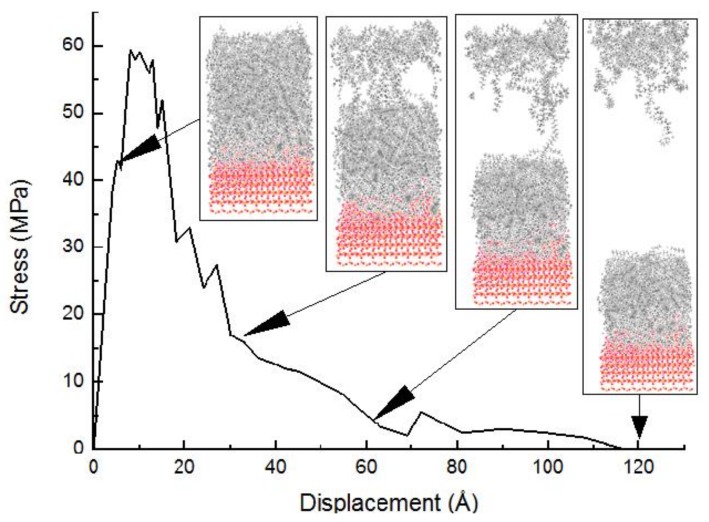
The tensile stress-displacement curve of the PE(76-40)/mS1 interface and snapshots of tensile simulation at different displacements.

The tensile stress-displacement curve of PE(76-40)/mS1 deformed at 300 K is exhibited in Figure 6. The displacement at the maximum stress in Figure 6 is 8 Å, which is less than that in Figure 5. The reason is that the coupling agent increases the bonding at the interface, which restricts the movement of chains. Meanwhile, the shorter chain length reduces the corresponding displacement at the maximum stress. The maximum stress in Figure 6 is 59.4 MPa, which is also less than that in Figure 5. The longer chain increases the entanglement. Therefore, it enhances the tensile strength. In Figure 6, the voids appear near the upper surface of the PE bulk. The reason is that the lower part of the PE bulk sticks to the surface of S by coupling agents and the damage does not occur. The debonding among PE chains occurs. These voids become bigger and coalesce when the elongation is 30 Å. The separation among the PE chains is due to the strong interaction between the PE chains and coupling agents. Hence, the PE bulk is weaker than the interface. The column in Figure 6 is much shorter than that in Figure 5 because of the shorter chain length of PE. As the separation increases to 60 Å, the void increases further and the column breaks. The polymer chains are pulled out from their starting configurations. The failure caused by the breakdown of nonbonded interactions in the PE bulk results in the cohesive failure mode at the displacement of 116 Å. The failure process of PE/mS is similar to that of PE/S, while the remaining part of PE on the S surface is larger.

#### 3.2.2. Effect of Chain Length, Number of Chains and Coupling Agents

**Figure 7:**
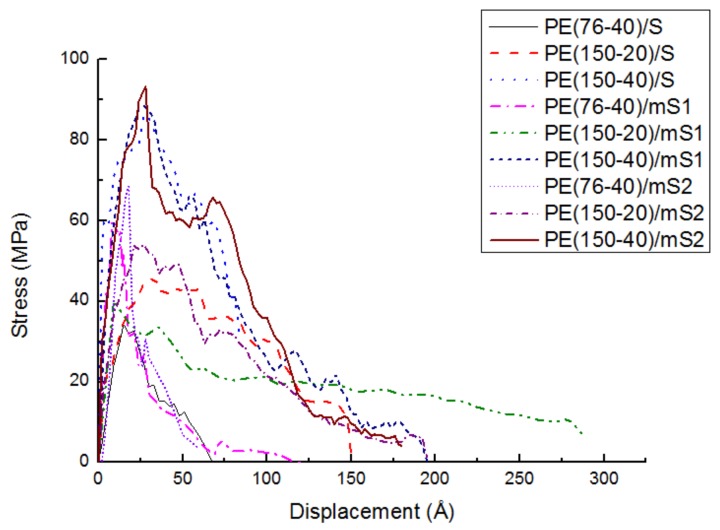
The effect of PE chain length, number and coupling agents on the tensile stress-displacement behavior of PE/S at 300 K.

The effect of PE chain length, number and coupling agents on the tensile stress-displacement simulation is studied at 300 K in Figure 7. The curves show large fluctuations, especially for chains with 150 carbon atoms. The maximum stress and the corresponding distance at the maximum stress in different cases are illustrated in Table 2. The maximum stress reaches 34.2 MPa and 86.3 MPa for PE(76-40)/S and PE(150-40)/S, respectively. The separation displacement is 15 Å and 27 Å, respectively, when the maximum stress occurs. It means that with a longer chain length, the maximum stress and corresponding displacement increase. The maximum stress reaches 42.8 MPa and 86.3 MPa for PE(150-20)/S and PE(150-40)/S, respectively. The separation displacement is 26 Å and 27 Å, respectively, when the maximum stress occurs. It means that with a larger chain number, the maximum stress and corresponding displacement also increase. The rise of separation displacement and maximum stress is caused by the entanglement. This behavior can be ascribed to increasing the chain tangling probability of the chains, which consequently improves the adhesion properties due to the greater energy dissipation. The lower chain length and number decrease the chain entanglement and the resistance of the polymer chains against deformation in the interface tensile process.

Table 2 shows that the tensile strength of PE/mS is greater than that of PE/S. Further, the tensile strength of the PE/mS2 is greater than that of the PE/mS1. The reason lies in that the PE can interact with A174 and A2783 better than S. The chemical bonds between A2783 and PE are stronger than the nonbonded interactions between A174 and PE. The stress transferring path is more complex due to the coupling agents. The PE/mS2 has the maximum displacement at the maximum stress in each group since the A2783 molecule is longer than the A174 molecule. This separation behavior in the PE/S system significantly differs from the separation behavior in the PE/graphite system presented in the previous study [9]. For the PE/graphite, the final separation occurs near the interface for the small systems or clearly at the interface for the large systems. However, for the PE/S, none of the separation occurs at the interface in our simulations, where the traction-separation responses show a ductile behavior because the separation occurs inside the polymer or near the interface. 

#### 3.2.3. Density Profile Analysis

**Figure 8:**
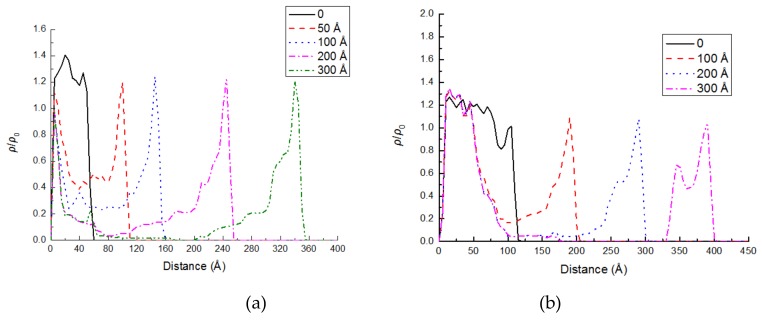
The reduced density of the chains versus the distance from silica surface at the various displacements of PE top rigid layer in (**a**) PE(150-40)/S interface; (**b**) PE(150-40)/mS1 interface.

Figure 8a,b show the density profile of PE(150-40)/S and PE(150-40)/mS1 interface at different stretching distances, respectively. In Figure 8a, the density profile reaches to zero value while the peaks in the vicinity of the S layer are very narrow when the displacement is 300 Å. 

This pattern corroborates the pulling of the chains from the layer adhered to the silica and exhibits the adhesive failure. The strong interactions at the interface make many chain segments remain on the silica surface and exhibit the cohesive failure in Figure 8b. However, the constant density value in the modified interface is smaller than that of the unmodified PE(150-40)/S interface. This behavior illustrates that the chains can detangle further by relying on the stronger interface. More PE are remained on the surface of S due to stronger coupling agents.

#### 3.2.4. Potential Energy Evolutions

**Figure 9:**
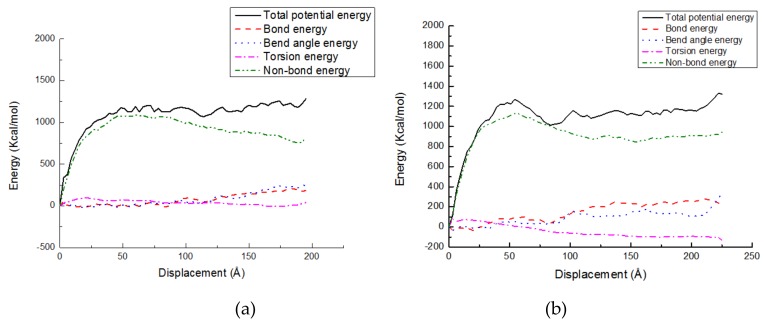
Potential energy-displacement curves of the (**a**) PE(150-40)/S interface; (**b**) PE(150-40)/mS1 interface.

The energy changes are exhibited in various potential energy contributions, such as bond stretching, angle bending, dihedral angle torsion, and nonbonded energies, versus tensile displacement for the PE(150-40)/S and PE(150-40)/mS1 interfaces at the temperature of 300 K in Figure 9a,b, respectively. In Figure 9a, the bond energy does not change greatly, while it increases with the distance obviously in Figure 9b, which illustrates that the bond length increases largely in PE(150-40)/mS1 interfaces. This means the A174 can drag the PE and stop it from moving away from S by extending the chain. In Figure 9b, the torsion energy decreases with the distance, which implies that the dihedral angles are not initially at their equilibrium state and move backward to their equilibrium states during the deformation process. It occurs since that the entanglement in PE(150-40)/mS1 is greater than that in PE(150-40)/S. The nonbonded energy sharply increases due to breaking the nonbonded interactions between the chains during the deformation of the systems. In the tensile deformation of equilibrated PE chains, the nonbonded energy plays a crucial role. 

## 4. Conclusions

The tensile failure and adhesion properties of the PE/S and PE/mS interfaces are studied at the microscale. The influence of the PE chain length, the PE chain number and coupling agents is considered during the tension process. It is observed that the stress versus displacement curves exhibit an elastic region followed by a yielding, post-yield and failure region in tensile cases. Increasing the number and the length of the PE chains considerably improves the adhesion strength and corresponding displacements in the tensile cases. In addition, the adhesion properties of the modification of S surface with A174 and A2783 coupling agents are strengthened. The failure process, potential energy evolutions and density profile are analyzed at microscale. Different failure modes are observed. The thermodynamic work of adhesion for different PE/mS interfaces increases due to the coupling agents. The present simulation study can help us to understand and tune the interfacial behavior between concrete and PE. It is expected to contribute to restricting the crack width, and increase the autogenous healing effect of ECC indirectly. 

## Figures and Tables

**Table 1:** Thermodynamic work of adhesion for different PE/S interfaces.

System	Work of Adhesion [mJ m^−2^]
PE(76-40)/S	93.14
PE(76-40)/mS1	3205.253
PE(150-20)/S	163.3887
PE(150-20)/mS1	3235.154
PE(150-40)/S	335.7157
PE(150-40)/mS1	3217.675

**Table 2:** The tensile strength and the corresponding distance at the maximum stress of the interfaces calculated at 300 K.

System	Adhesion Strength (MPa)	Distance (Å)
PE(76-40)/S	34.2	15
PE(76-40)/mS1	59.4	8
PE(76-40)/mS2	68.6	18
PE(150-20)/S	42.8	26
PE(150-20)/mS1	43	9
PE(150-20)/mS2	54.0	27
PE(150-40)/S	86.3	27
PE(150-40)/mS1	88.5	27
PE(150-40)/mS2	93.2	28
